# High-dose-rate brachytherapy lowers travel burden for men with localized prostate cancer compared with external beam radiation

**DOI:** 10.3389/fruro.2025.1598726

**Published:** 2025-06-30

**Authors:** Catherine Liu, Hongmei Yang, Kevin Bylund, Michael Cummings, Hong Zhang

**Affiliations:** ^1^ University of Rochester School of Medicine and Dentistry, Rochester, NY, United States; ^2^ Department of Biostatistics & Computational Biology, University of Rochester Medical Center, Rochester, NY, United States; ^3^ Department of Radiation Oncology, University of Rochester Medical Center, Rochester, NY, United States

**Keywords:** prostate cancer, high-dose-rate brachytherapy, external beam radiation therapy, patient travel burden, access to care

## Abstract

**Objective:**

There are many treatment options for localized prostate cancer, including external beam radiation therapy (EBRT), stereotactic body radiation therapy (SBRT), and prostate brachytherapy (BT). This study aimed to compare the travel burdens of high-dose-rate brachytherapy (HDR-BT) at our BT center and EBRT or SBRT if administered close to home.

**Materials and methods:**

This single-institution retrospective cohort study included 69 patients who had HDR-BT monotherapy for their prostate cancer from August 2017 to December 2022. The travel burden for HDR-BT monotherapy was estimated using Google Maps by measuring the distance from each patient’s home address to our BT center. The total travel burden was calculated by multiplying the number of treatment fractions required for each modality by the roundtrip travel distance between the home and the treatment facility. Treatment toxicity was evaluated using the Expanded Prostate Index Composite for Clinical Practice (EPIC-CP) questionnaire.

**Results:**

The median age of the 69 patients was 67 years. The mean distance from home to the BT center was 37.4 mi, while the mean distance to the nearest radiation facility was 8.3 mi. The mean total travel distance for HDR-BT was 150 mi, while those for EBRT and SBRT were 463 and 83 mi, respectively. HDR-BT resulted in a mean travel burden reduction of 313 mi compared with EBRT. The EPIC-CP scores indicated minimal posttreatment toxicity, with most patients reporting stable or improved symptoms.

**Conclusion:**

HDR-BT monotherapy significantly reduces the travel burden compared with EBRT for localized prostate cancer, with minimal treatment-associated toxicity. Increasing the availability of BT centers could further alleviate the travel burden. Alternatively, providing transportation support could improve access to care.

## Introduction

Prostate cancer is the most common malignancy after skin cancer and the second leading cause of cancer death among men in the United States. The American Cancer Society estimated 313,780 new cases and 35,770 deaths from prostate cancer in the United States in 2025 (1). Patients in this country face numerous barriers to their cancer care, including financial, social, communication, and logistical challenges. These barriers, unfortunately, have a significant impact on patient outcomes. Studies have shown that patients with prostate cancer in high-poverty neighborhoods have a higher risk of cancer death compared with those in wealthy areas (2). Therefore, it is important to consider both the efficacy of and the access to treatments offered to address healthcare disparities.

One significant barrier to cancer care is the travel burden placed on patients, particularly those in rural or disadvantaged areas. Traveling long distances to receive care can lead to increased costs, lost wages, delayed diagnosis, and poor outcomes (3). The travel time also impacts a patient’s treatment choice, with one study showing it as an independent predictor for the selection of prostate cancer treatment modality (4).

For non-metastatic prostate cancer, curative-intent treatments include surgery and radiation, with or without androgen deprivation. There are two types of radiation: external beam radiation therapy (EBRT) and brachytherapy (BT). EBRT delivers radiation generated by a linear accelerator (LINAC), whereas BT involves placement of radioactive sources either permanently (low-dose-rate or LDR) or temporarily (high-dose-rate or HDR) inside of the tumor target. BT can be used alone or in combination with EBRT, depending on the prostate cancer’s risk grouping (5–8). For patients with low- or intermediate-risk prostate cancer, BT may be used alone as a monotherapy (7). In addition, BT offers a substantial time advantage over EBRT, often requiring only one or two visits compared with approximately 28 trips for EBRT. Recent technological advancements have made stereotactic body radiation therapy (SBRT), which is an ultra-hypofractionated EBRT, an alternative treatment option that reduces treatment to just five trips. However, the use of BT has declined over time from 17% to 8% among patients with prostate cancer (9, 10). Despite declining usage, HDR-BT offers a compelling option with its high effectiveness in cancer control and comparable, if not fewer, side effects compared with surgery or external beam radiation, with no radiation exposure to the providers and staff compared with permanent BT (5, 7, 8, 11–25). Multiple factors have impacted the decline of BT, including less reimbursement based on our current fee-for-service model (26) and limited resident training (27).

In this study, we aimed to compare the travel burdens of HDR-BT and EBRT or SBRT for localized prostate cancer treated at our institution (28). The travel times of patients receiving HDR-BT as monotherapy were evaluated and then compared to hypothetical travel times if they had chosen EBRT or SBRT at the facility closest to their home. The results of this study provide insights into the challenges patients with prostate cancer face during treatment so that appropriate support can be provided for them.

## Methods

This single-institution, retrospective cohort study examined the travel burden of 69 patients who had HDR-BT monotherapy for prostate cancer at our BT center from August 2017 to December 2022. Each patient received two fractions of BT, at 13.5 Gy per fraction, 1 week apart. Prostate-specific antigen (PSA) values were collected at posttreatment follow-up visits to assess treatment outcomes.

The travel burden for HDR-BT monotherapy was estimated by measuring the distance from each patient’s home address to our BT center using Google Maps. If multiple routes were suggested by Google Maps, the route with the shortest travel time was selected. Similarly, the distance between each patient’s home address and the nearest EBRT facility was measured. The total travel burden was calculated by multiplying the number of treatment fractions required for each modality by the roundtrip travel distance between the home and the treatment facility. All patients received two treatment fractions for HDR-BT monotherapy. For EBRT, 28 fractions were used as this is the standard treatment regimen for low- and intermediate-risk prostate cancer at our institution. It was assumed that all of the EBRT facilities also offered SBRT, which required only five fractions.

The Expanded Prostate Index Composite for Clinical Practice (EPIC-CP) questionnaire was used to evaluate treatment toxicity. EPIC-CP covers five symptom categories: urinary incontinence, urinary irritation/obstruction, bowel, sexual, and vitality/hormonal. Patients completed the EPIC-CP before treatment (baseline; 57% of the patients completed the survey) and at 3, 6, 12, 24, 36, and 48 months post-therapy (17%, 23%, 26%, 22%, 13%, and 10% of the patients completed the survey, respectively). The overall survival (OS) and disease-free survival (DFS) were estimated.

### Statistical methods

Continuous variables were summarized using means and medians with corresponding data ranges. The within-subject travel burdens were compared using a paired *t*-test and confirmed by nonparametric analogy of the signed-rank test. The survival rate was estimated non-parametrically with the Kaplan–Meier statistical method. Statistical significance was defined at a *p* ≤ 0.05. All statistical analyses were conducted using version 9.4 of the SAS System for Windows (SAS Institute Inc., Cary, NC, USA).

## Results

### Patient characteristics

A total of 69 patients received HDR-BT monotherapy for prostate cancer at the BT center between August 2017 and December 2022 (**Table 1**). The median age was 67.9 years(range, 52–82 years), with the majority of patients identifying as white (95.7%). Most patients had either the favorable intermediate (69.6%) or the unfavorable intermediate (23.2%) risk disease. There were 64 (93%) patients who had a Gleason score of 6 or 7 (4 + 3 or 3 + 4) disease, while 60 (87%) patients had cT1c disease. The mean and median pretreatment PSA levels were 6.7 and 5.8, respectively (range, 1.39–22.38). The median follow-up was 32.9 months (range, 3.5–77.2 months). The mean and median posttreatment PSA nadir levels were 0.59 and 0.39, respectively (range, <0.02–3.78). Only one patient had biopsy-proven recurrence and underwent salvage HDR-BT.

**Table 1:** Patient and cancer characteristics (*n* = 69).

Age (years)		
Median	67.0	
Average	67.9	
Risk grouping		
Low	16	23.2%
Favorable intermediate	48	69.6%
Unfavorable intermediate	4	5.8%
High	1	1.4%
PSA		
Median	5.8	
Average	6.7	
Gleason score		
3 + 3	20	29.0%
3 + 4	44	63.8%
4 + 3	3	4.3%
4 + 4	2	2.9%
T stage		
1c	60	87.0%
2	9	13.0%
Race		
White	66	95.7%
Black	3	4.3%

### Travel burden of HDR-BT

**Figure 1:**
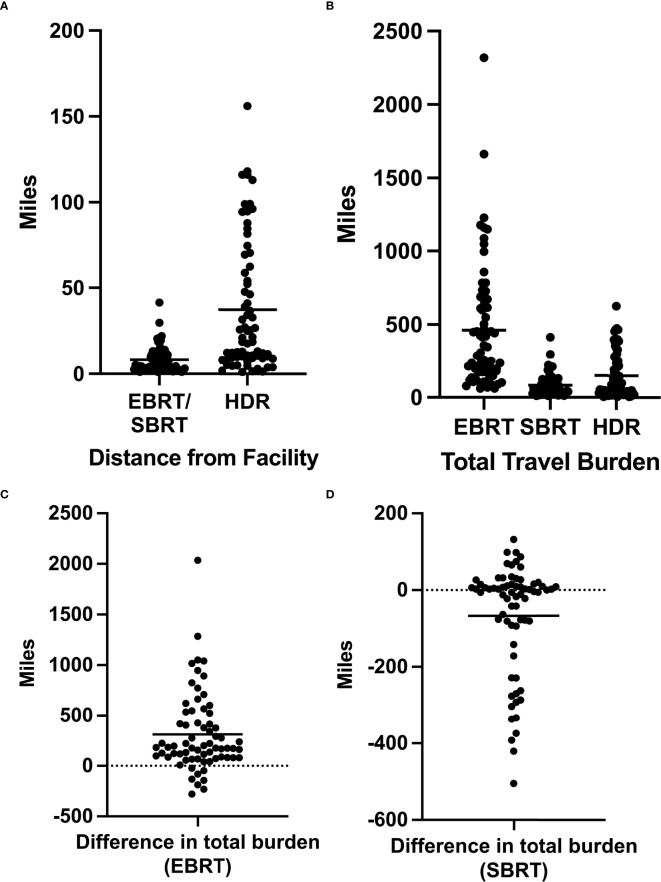
**(A)** Distance in miles between a patient’s home and the facilities offering external beam radiation therapy (EBRT)/stereotactic body radiation therapy (SBRT) or high-dose-rate brachytherapy (HDR-BT). **(B)** Total travel burden as defined by the total miles traveled over the entire duration of treatment for EBRT, SBRT, and HDR-BT. **(C)** Difference in the total travel burden for each patient as calculated by the total EBRT travel burden − total HDR-BT travel burden. **(D)** Difference in the total travel burden for each patient as calculated by the total SBRT travel burden − total HDR-BT travel burden. *Horizontal bars* represent average values.

In our cohort of patients, the mean distance between home and the BT center was 37.4 mi (range, 1.1–156 mi). For this cohort, the mean distance between home and the nearest EBRT facility was 8.3 mi (range, 1.1–41.4 mi) (**Figure 1A**). Considering the number of roundtrips required for treatment completion, the mean total distance traveled for our cohort of patients treated with HDR-BT was 150 mi (range, 4.4–624 mi) (**Figure 1B**). If the same group had chosen EBRT treatment at their nearest facility, the mean total distance traveled would have been 463 mi (range, 61.6–2,318 mi) (**Figure 1B**). For this same group, if treated with SBRT, the mean total distance traveled would have been 83 mi (range, 11–413.9 mi) (**Figure 1B**).

The mean difference in the total travel burden between EBRT and HDR-BT monotherapy was 313 mi (**Figure 1C**). Of the 69 patients, 61 (88%) had a decreased total travel distance by choosing HDR-BT over EBRT, while 8 (12%) patients had an increased total travel distance. Patients with decreased total travel distance saved an average of 372 mi (range, 9.2–2,036 mi). Those with increased total travel distance saw a mean increase of 141 mi (range, 24–274 mi).

The mean difference in the total travel burden between HDR monotherapy and SBRT was 64 mi (range, 193–505 mi) (**Figure 1D**). Of the 69 patients, 36 (52%) had an increased travel distance by choosing HDR-BT over SBRT (mean of 157 mi), 33 (48%) had a decreased travel distance (mean of 37 mi), and one had the same travel distance. For patients with increased total travel burdens, the mean distance between home and the BT center was 59 mi (11–156 mi).

### HDR-BT posttreatment toxicities

Based on the EPIC-CP reports, patients did not experience worsening genitourinary (GU), gastrointestinal (GI), and sexual symptoms after HDR-BT treatment compared with the baseline.

**Figure 2:**
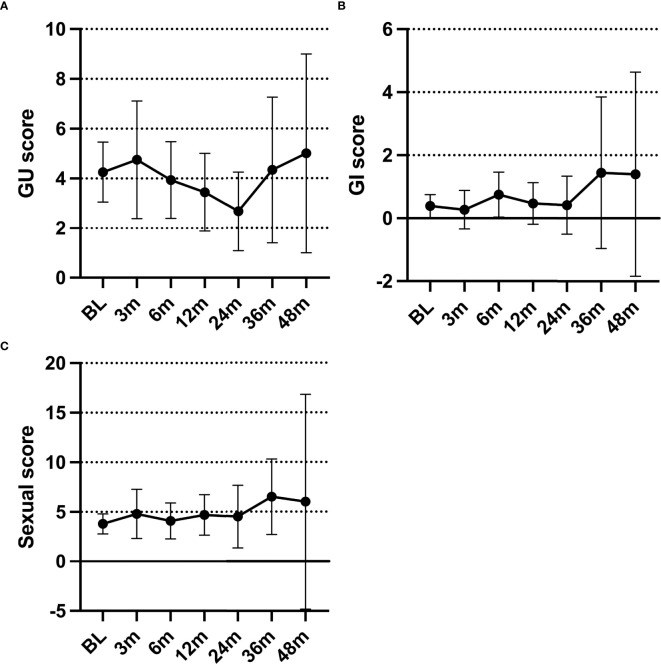
**(A–C)** Average Expanded Prostate Index Composite for Clinical Practice (EPIC-CP) genitourinary **(A)**, gastrointestinal **(B)**, and sexual **(C)** scores over time.

The mean EPIC-CP GU scores initially increased at 3 months (4.75), then steadily declined over the first 2 years (2.67 at 24 months) (**Figure 2A**). After 2 years, the mean EPIC-CP GU scores increased again. At the last review, 87% of the patients had an EPIC-CP GU score that was equal to or lower than their baseline, with only five patients reporting an EPIC-CP GU score higher than the baseline.

Overall, the EPIC-CP GI scores were low (**Figure 2B**). Most patients (62%) reported a baseline EPIC-CP GI score of 0. The mean EPIC-CP GI scores never surpassed 2 at any point. Only five patients reported an increase in their posttreatment EPIC-CP GI score from the baseline.

The mean EPIC-CP sexual scores showed minimal change within the first 2 years (range, 3.79–4.8) (**Figure 2C**). After the first 2 years, the mean EPIC-CP sexual scores increased. At the last review, 76% of the patients had an EPIC-CP sexual score that was equal to or lower than the baseline, while seven patients reported a score higher than the baseline.

### HDR-BT outcomes

**Figure 3:**
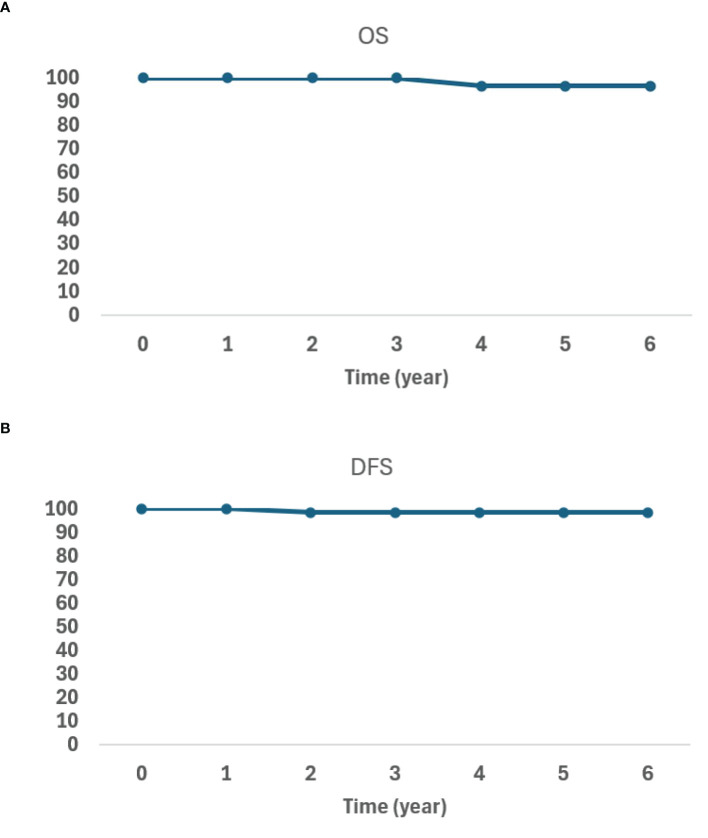
Kaplan–Meier overall survival (OS) **(A)** and disease-free survival (DFS) **(B)** curves.

After the median follow-up of 32.9 months, one patient died from pneumonia, and another had a biopsy-proven prostate-only recurrence. The patient with a biopsy-proven recurrence initially presented with a PSA of 4.87 and a Gleason score of 4 + 3 = 7/grade group 3 disease. The patient declined androgen deprivation therapy (ADT). His PSA nadir was 0.17, but increased to 1.37 at 54 months post-BT. Restaging prostate-specific membrane antigen (PSMA) PET and MR prostate showed imaging evidence of recurrence within the prostate. Prostate biopsy showed Gleason 4 + 3 = 7/grade group 3 disease only at the imaging-visible site. He underwent salvage focal HDR-BT targeting the biopsy-proven lesion. At his last follow-up, 17 months post-treatment, his PSA was 0.43. The OS and DFS data are shown in **Figures 3A, B**.

## Discussion

In this study, we compared the travel burdens of patients with prostate cancer choosing HDR-BT monotherapy *versus* EBRT and SBRT for treatment. The findings indicate that patients receiving HDR-BT monotherapy experienced significantly less travel burden compared with those treated with EBRT. However, for patients living more than 59 mi from the BT center, SBRT resulted in less overall travel burden. In addition, our cohort of patients treated with HDR-BT demonstrated excellent oncological outcomes with minimal treatment-associated toxicities.

Our analysis revealed that the patients in our cohort lived substantially further from our BT center than from EBRT facilities. This underscores the scarcity of clinics offering BT, likely contributing to the lower number of patients receiving BT for prostate cancer, as supported by other studies (29).

We employed a novel approach using a single patient cohort as its control to compare the travel distances. However, we acknowledge several limitations to this approach. We assumed that the closest EBRT facility to the patient’s home would be the chosen treatment center. In reality, multiple factors may influence the patient’s choice, including provider preferences and insurance coverage. Another limitation was our assumption that the EBRT facilities also offered SBRT.

While SBRT and HDR-BT both deliver ablative radiation doses, there is no level 1 evidence comparing these two treatment modalities. HDR-BT now has reported outcomes with approximately 10 years of median follow-up (18). However, long-term follow-up is needed to establish the long-term effectiveness and safety profile of SBRT.

Our patient cohort was predominantly white, which does not reflect the typical prostate cancer population demographics near our practice, suggesting a bias in the patient population. The lack of racial diversity may also indicate additional barriers to accessing BT. Future studies should investigate the factors influencing patients’ decision to choose BT as their treatment modality.

Our study had a short follow-up time. In addition, many patients did not fill out the EPIC-CP questionnaire consistently at follow-up visits, limiting the accuracy of our toxicity profile. Despite these limitations, limited toxicity and excellent outcomes were observed, consistent with other studies on the toxicity and efficacy of HDR-BT in prostate cancer treatment (13, 17, 18, 20, 23, 30–32).

In conclusion, despite its invasive nature, HDR-BT is an appealing option for patients with localized prostate cancer, especially for those with issues regarding proximity to a healthcare facility. Increasing the number of centers offering BT or transportation support could help address the disparity in prostate cancer care.

## Data Availability

The raw data supporting the conclusions of this article will be made available by the authors, without undue reservation.
